# LOUPE: observing Earth from the Moon to prepare for detecting life on Earth-like exoplanets

**DOI:** 10.1098/rsta.2019.0577

**Published:** 2021-01-11

**Authors:** D. Klindžić, D. M. Stam, F. Snik, C. U. Keller, H. J. Hoeijmakers, D. M. van Dam, M. Willebrands, T. Karalidi, V. Pallichadath, C. N. van Dijk, M. Esposito

**Affiliations:** ^1^ Aerospace Engineering, Delft University of Technology, Kluyverweg 1, 2629 HS, Delft, The Netherlands; ^2^ Leiden Observatory, Leiden University, PO Box 9513, 2300 RA, Leiden, The Netherlands; ^3^ Center for Space and Habitability (CSH), University of Bern, Gesellschaftsstrasse 6 (G6), 3012 Bern, Switzerland; ^4^ Department of Physics, UCF, 4111 Libra Drive, Physical Sciences Building 430, Orlando, FL, USA; ^5^ cosine Remote Sensing, Oosteinde 36, 2361 HE Warmond, The Netherlands

**Keywords:** LOUPE, spectropolarimetry, Earth as an exoplanet

## Abstract

LOUPE, the Lunar Observatory for Unresolved Polarimetry of the Earth, is a small, robust spectro-polarimeter for observing the Earth as an exoplanet. Detecting Earth-like planets in stellar habitable zones is one of the key challenges of modern exoplanetary science. Characterizing such planets and searching for traces of life requires the direct detection of their signals. LOUPE provides unique spectral flux and polarization data of sunlight reflected by Earth, the only planet known to harbour life. These data will be used to test numerical codes to predict signals of Earth-like exoplanets, to test algorithms that retrieve planet properties, and to fine-tune the design and observational strategies of future space observatories. From the Moon, LOUPE will continuously see the entire Earth, enabling it to monitor the signal changes due to the planet’s daily rotation, weather patterns and seasons, across all phase angles. Here, we present both the science case and the technology behind LOUPE’s instrumental and mission design.

This article is part of a discussion meeting issue ‘Astronomy from the Moon: the next decades (part 1)’.

## 1. Introduction

Since the first discoveries of planets orbiting other stars in the 1990s, exoplanetary research has expanded explosively. Today, we know of more than 4000 such worlds, ranging from gas giants more massive than Jupiter to rocky, terrestrial-type planets considered to be candidates for harbouring life. Although we now know planets orbiting other stars are not uncommon, the occurrence rate of Earth-like planets in the habitable zone of Sun-like stars remains a highly debated topic. Statistical analysis of existing exoplanetary catalogues has shown that somewhere between 2% and 60% of Sun-like stars may harbour planets similar to the Earth (or super-Earth) in their habitable zones [1]. The exoplanetary catalogues are being expanded daily through space missions such as NASA’s TESS (Transiting Exoplanetary Survey Satellite), which is expected to detect more than 14 000 exoplanets, of which over 2100 will be smaller than 4 Earth-radii [2]; whereas ESA’s upcoming PLATO (PLAnetary Transits and Oscillations of stars) mission will focus on habitable worlds around Solar-type stars, aiming to yield between 6 and 280 Earth analogues out of approximately 4600 total detections [3]. Knowing that Earth-like rocky exoplanets might be more common than previously thought, the next step is investigating their atmospheres, surfaces and biomarkers. Although transit spectroscopy is a well-established method for characterizing gas giants, transit signals of the thin atmospheres of Earth-like exoplanets around Sun-like stars are undetectable with present technology. A significant upcoming technological milestone for astronomy will be to achieve direct imaging, in which a planet’s (reflected) starlight is observed separately from the light of its host star. Resolving the planet from its star will offer an opportunity to investigate its properties via spectral flux and polarization measurements. Furthermore, direct imaging will enable non-transiting planets to be detected and characterized.

The most reliable benchmark for characterizing Earth-like exoplanets is, naturally, the Earth. By placing the observer at a distance such that the Earth appears as an unresolved ‘pale blue dot’, we may simulate the observation of Earth as an exoplanet. In this single ‘dot’, all the spectropolarimetric information from sunlight reflected off of Earth’s oceans, continents, biomarkers and clouds is integrated into a spatially unresolved point. If we can reliably extract this information from the unresolved signal and reverse-engineer the properties of the Earth as we know it, we will have developed a powerful tool for characterizing exoplanets, including their oceans, continents, atmospheric composition and life signatures, even if we are unable to spatially resolve them.

The Earth is already continuously being observed by remote-sensing satellites, which monitor, e.g., atmospheric trace gas concentrations, crop health and weather patterns. Apart from the fact that there are currently no Earth remote-sensing satellites with polarimetric capabilities,^1^ such Low Earth Orbit observations typically have their field-of-view limited to localized portions of the Earth’s surface, and not the entire Earth’s disc. A mosaic of such observations does not realistically represent the instantaneous single-pixel view of Earth, because the individual segments vary in terms of local time and weather conditions, and the distribution of local illumination and viewing geometries is very different from the distribution when the Earth is viewed from afar. In particular, most satellites have a nadir viewing direction, and are in sun-synchronous orbits, observing a given location on the Earth at more or less the same time of day. Especially the polarization is very sensitive to the illumination and viewing angles [4]. Even satellites locked in geostationary orbit would not provide us with complete insight, as they only observe a single hemisphere, thus missing out on the variations due to the daily rotation.

Various reasons make observing the Earth from the Moon, from a Lunar orbit, or the Earth-Moon L1-point, rather than a low Earth orbit, crucial to the experiment:

1. The Moon is sufficiently far away to allow a spatially unresolved view of the whole Earth.
2. For a lander on the Lunar surface, the Earth is always visible in a confined area in the sky.^2^
3. From the Moon, the Earth can be observed at all phase angles^3^ during a month.
4. From the Moon, the Earth’s daily rotation can be captured.

The last one provides a view of the entire Earth, and allows detecting changes in Earth’s spectropolarimetric signals as continents and oceans rotate in and out of view. Observations covering several months could reveal seasonal changes. In the light of these advantages, we propose the Lunar Observatory for Unresolved Polarimetry of Earth (LOUPE) [5], a compact and small spectropolarimeter based on pioneering liquid crystal polarization optics, to accompany an orbiting, landing or roving mission on the near side of the Moon. LOUPE’s tentative instrument design is presented in §4, and the performance of a previous design iteration was validated in [6].

The main driver of LOUPE is to perform a long-term observing campaign of the Earth as if it were a spatially unresolved exoplanet, both in flux and polarization, in order to provide the ongoing search for Earth-like exoplanets with the benchmark of an archetypal Earth. Another approach to obtain such flux and polarization data is through the so-called ‘Earthshine’ observations [7–10], where ground-based telescopes are used to search for back-scattered light of the Earth on the shadowed crescent of the lunar disc. Although some spectral features of the Earth’s flux were reported, such as the O_2_-A band, the Vegetation Green Bump and Red Edge (VRE), this method is severely hampered by unknown depolarization effects of the reflection of polarized light by the Lunar surface, degradation of the signal as it re-enters the Earth’s atmosphere to reach the observer, and the severe difficulties in monitoring the daily rotation and a broad range of phase angles. LOUPE would eliminate these problems by observing from the Moon itself, creating a dedicated spectropolarimetric observing platform with superior science return.

LOUPE’s aim is to pioneer spectropolarimetry as a uniquely qualified tool for exoplanet characterization. For ground-based telescopes, polarimetry makes it possible to differentiate between the reflected flux of a planet and the overpowering flux of its parent star [11] even when direct detection is not possible from intensity alone, enabling us to find exoplanets which would otherwise be lost in the stellar glare. This ability stems from the fact that sunlight and—more generally—light of Solar-type stars can be assumed to be unpolarized when averaged over the stellar disc, whereas the light scattered in a planetary atmosphere and/or reflected by a planetary surface will generally become polarized (up to 10%.) Thus, measuring the polarized flux of a planet can be used to enhance the contrast between the two. Future space telescopes like HabEx/LUVOIR aim to deliver the intrinsic 10^−10^ contrast to directly image an Earth orbiting a Solar-type star, and then polarimetry can be applied to further characterize the planet. As Solar System observations have indicated [12], the phase angle dependence of the linearly polarized spectrum of a planet is highly sensitive to atmospheric constituents and clouds, as well as surface features like vegetation, water, ice, snow or deserts (see §2.) Therefore, the main advantage of spectropolarimetry is the ability to deliver unambiguous characterization of exoplanets, breaking the retrieval degeneracies arising from flux measurements alone. In this way, polarimetry promises to reveal not only a plethora of new worlds, but also a plethora of new geomorphologies and biospheres.

One of our main goals of monitoring the Earth from afar is to gather benchmark data to test the radiative transfer codes which are being used to compute signals of rocky exoplanets [13,14]. Such signals are crucial for the design of future (space) telescopes dedicated to the characterization of Earth-like exoplanets and for the development of algorithms to retrieve exoplanet characteristics [15]. Previous attempts to study Earth as an exoplanet involved serendipitous measurements from deep space instruments used outside their intended mode of operation, e.g. the Galileo [16], Deep Impact [17], Venus Express [18], DSCOVR [19–21] and LCROSS [22] space probes. With their limited coverage, these experiments are unsuited to study the whole phase angle range and to achieve full global coverage, nor could they measure polarization, thus falling short of a complete and thorough characterization of the Earth’s disc-integrated signal. LOUPE’s monitoring of the total flux that the Earth reflects would also be valuable for climate research, as this reflected flux and its spectral and temporal variations give insight into the amount of Solar energy that the Earth absorbs over time. The polarization signal of the Earth as a whole could provide new information on high-altitude aerosol particles that contribute to the Earth’s radiation balance by reflecting incoming sunlight and by heating up their ambient environment, as well as playing important roles in chemical reactions [23,24].

Section 2 discusses interesting features in the Earth’s flux and polarization signals. Science requirements and the resulting instrument requirements and goals are presented in §3, and an overview of LOUPE’s instrument design in §4. Conclusion is presented in §5.

## 2. Earth’s flux and polarization signals

In the absence of real spectropolarimetric data of the spatially unresolved Earth, we use numerical simulations for the design of LOUPE. A key part of LOUPE’s mission is to provide benchmark data for the improvement and refinement of such numerical simulations of exoplanet signals.

We describe light as a Stokes (column) vector [4]:

2.1 F=[F,Q,U,V],

with *F* the total flux, *Q* and *U* the linearly polarized fluxes, and *V* the circularly polarized flux (all in W m^−2^ nm^−1^). Fluxes *Q* and *U* are defined with respect to a reference plane, for which we use the planetary scattering plane, i.e. the plane through the centres of the Sun, the Earth and the observer, which in our case is LOUPE on a Lunar orbiter or lander. The degree of (linear) polarization of the light is defined as

2.2 $$P_{L}=\frac{\sqrt{Q^{2}+U^{2}}}{F}.$$

The angle of polarization, *χ*, is also defined with respect to the reference plane: tan 2*χ* = *U*/*Q*, where the sign of *χ* is such that 0 ≤ *χ* < *π* and that it equals that of *Q* [4].

**Figure 1.**
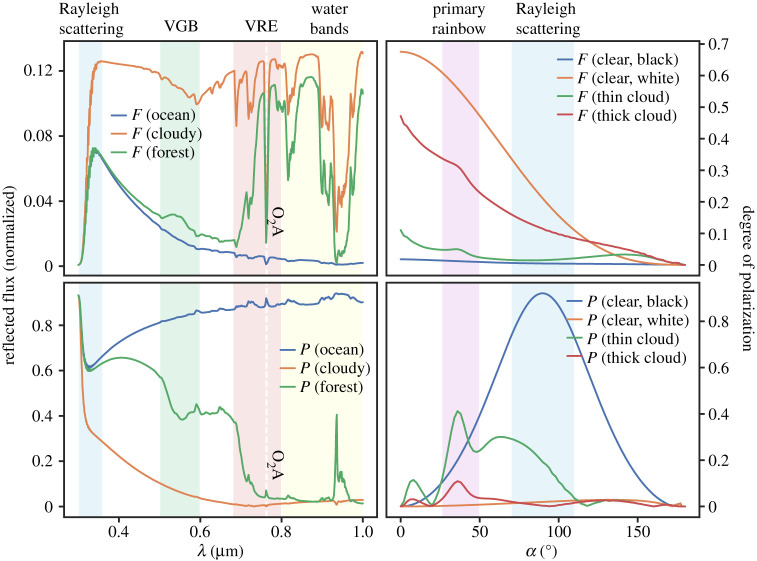
Simulated reflected flux and polarization at a phase angle *α* of 90° as functions of the wavelength (left, from [13]) and as functions of *α* (right, from [26]) for horizontally homogeneous planets with varying surface properties and cloud covers. The fluxes have been normalized such that at *α* = 0, they equal the planet’s geometric albedo. Apart from absorption by oxygen (O_2_), absorption by ozone and water-vapour is included. (Online version in colour.)

We compute **F** (equation (2.1)) of the visible and illuminated disc of a model Earth at a given phase angle *α* and wavelength λ by dividing the disc into pixels with specific surface–atmosphere models (e.g. ocean-cloudy, forest-clear, desert-clear), computing the reflected Stokes vector for each pixel using an adding-doubling radiative transfer algorithm [13,14,25], and summing up the local vectors to obtain the disc-integrated, planetary Stokes vector. An example of the simulated flux and polarization for an unresolved planet is shown in figure 1.

**Figure 2.**
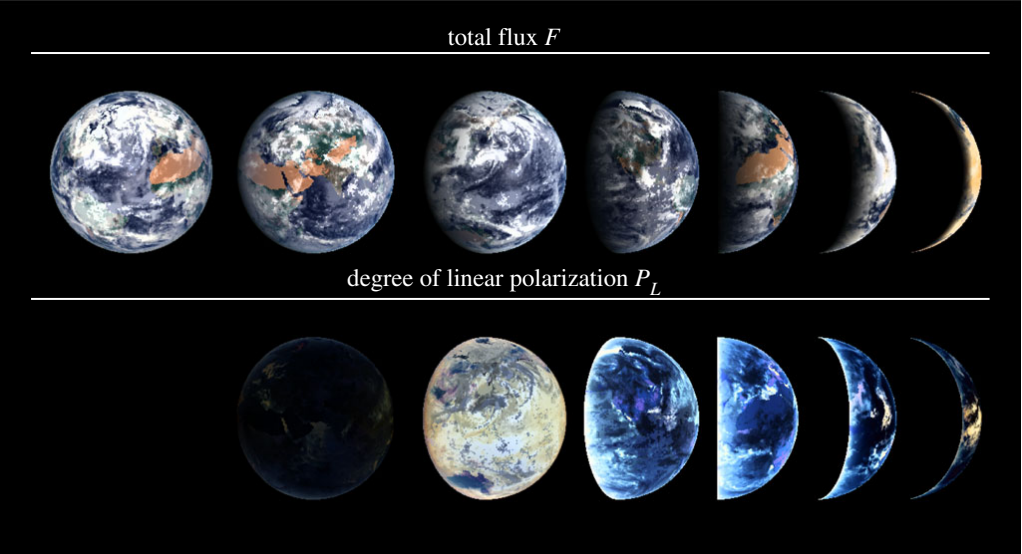
Computed *F* (top) and *P*_*L*_ (bottom) for the Earth at phase angles *α* starting with 0° on the left. An RGB-colouring scheme combined with a grey-scale was used to show both the spectral dependence and the absolute value of the reflected flux and the polarization. Note that at *α* = 0°, *P*_*L*_ is virtually zero. For these images, data described in [25] were used. (Online version in colour.)

Figure 2 shows the computed *F* and *P*_*L*_ (equation (2.2)) for a spatially resolved model Earth at several phase angles. Various features in *P*_*L*_ stand out. Firstly, at *α* = 0°, *P*_*L*_ is zero across the disc because of the symmetric, back-scattering geometry for each pixel. Secondly, clouds generally have low *P*_*L*_, and oceans with only Rayleigh-scattering gas above them, a high *P*_*L*_. Thirdly, at large phase angles, *P*_*L*_ is highest in the red, due to the glint on the ocean [27]. In the following subsections, we discuss some notable reflective properties of the planetary surface and atmosphere.

### (a) Continents and oceans

Owing to plate tectonics, the Earth’s surface is covered by continents and oceans. The continents are covered by various surface types, such as rocks, sand, snow and vegetation, each with characteristic (wavelength-dependent) albedos and bidirectional reflectance functions (i.e. the angular distribution of the reflected total and polarized fluxes). Rough surfaces reflect more or less isotropically and strongly depolarize the incident light. Generally, the higher the albedo of such surfaces, the lower *P*_*L*_, as the total flux increases but the polarized fluxes do not [13].

Ocean surfaces exhibit specular, Fresnel, reflection, which is anisotropic and polarizing. The glint of sunlight on water is a particularly striking feature, arising when the angles of incidence and reflection are equal. Waves influence the appearance of the glint: generally, the higher the wind velocity, the higher the waves, and the broader the glint pattern that is expected to appear on the disc. With LOUPE, we can investigate this relationship and the influence of other wave parameters, such as white caps and wave direction. Numerical results suggest that an ocean on a planet can be uniquely identified by a colour change of *P*_*L*_ of a planet at intermediate to large phase angles: only with an ocean, a planet will change from blue, through white, to red, with increasing *α*, when observed using polarimetry [27].

The numerically predicted effects of various reflecting surfaces on the planetary phase curve both in total flux and polarization can be observed by LOUPE.

### (b) Vegetation

Earth’s vegetation owes its green colour to a decrease in the absorption by chlorophyll (and thus an increase in the albedo) around λ = 500 nm. However, as is evident from figure 1, the most distinct spectral feature of vegetation is not this ‘Green Bump’, but the dramatic brightness just outside the human visible range, the ‘Vegetation Red Edge’ (VRE) [28]. Light-harvesting vegetation on exoplanets may have similar reflectance properties, as the VRE is hypothesized to limit excessive absorption of light at wavelengths where photosynthesis is inefficient. The VRE of exo-vegetation might cover different wavelengths than terrestrial vegetation, but strong spectral features of unknown geological or atmospheric origins could be worth investigating as possible biosignatures of alien vegetation.

A worthwhile exercise is to attempt to extract the VRE from the signal of a spatially unresolved Earth, as seen by LOUPE. Simulations suggest that the VRE ought to be detectable even through optically thick clouds, and that its signature in polarization is even more pronounced, as it is located in a wavelength region where the degree of polarization is highly sensitive to the surface albedo [13]. A tentative confirmation of the VRE in polarized light has been shown in Earthshine observations [7]. LOUPE strives to confirm and improve the detection with its beneficial vantage point on the Moon, without the strongly depolarizing influence of a reflection by the Lunar surface.

Vegetation has also been shown to exhibit a small, but unambiguous circular polarization signature, as a consequence of the homochiral configuration of organic matter [29]. A potential future ‘super-LOUPE’ could be upgraded to perform full-Stokes demodulation, for example building on the design of the Life Signature Detection polarimeter (LSDpol [30]; see also [31]), and retrieve the circularly polarized flux as an additional biomarker to be studied.

### (c) Clouds

Clouds generally decrease *P*_*L*_ because they add total flux but little polarized flux. However, the phase angle variation of *P*_*L*_ of a cloudy planet shows various interesting features, such as glories and, most notably, rainbows. Rainbows are a well-known optical phenomenon formed when light is scattered by airborne water droplets, such as rain droplets and also cloud droplets. In particular, the primary rainbow results from light rays which have undergone a single reflection inside spherical droplets. This rainbow exhibits dramatic peaks in both *F* and *P*_*L*_, as shown in figure 1. Due to the small size of terrestrial cloud particles (as compared to rain droplets), a cloudy Earth will only show a significant rainbow peak in *P*_*L*_ [32]. The rainbow angle depends on the particle composition: with water clouds, these peaks would appear around *α* = 40°, and could be used to identify the presence of liquid water clouds on exoplanets, even with small cloud coverage fractions and partly overlaid with ice clouds [32]. Clouds with different compositions, such as sulfuric acid clouds which are present on Venus, would produce rainbows at different phase angles [12]. Other numerical simulations show that the variability of *P*_*L*_ of an exoplanet would reveal the spatial distribution of clouds [14].

Observing the Earth’s clouds with LOUPE will give us a better understanding of the spectral and temporal variations in *F* and *P*_*L*_, which could be used to characterize the composition, spatial coverage and altitude of the cloud cover on exoplanets.

### (d) Oxygen and trace gases

**Figure 3.**
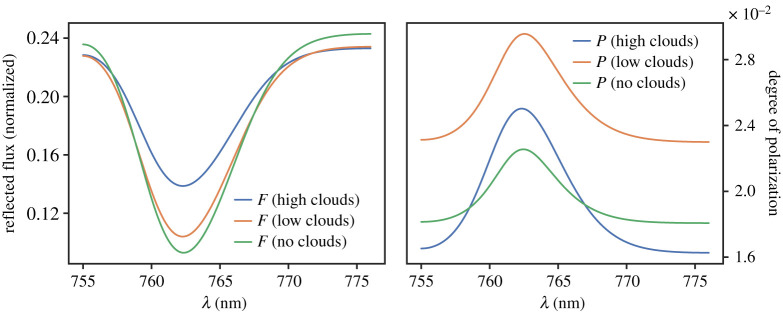
Total flux *F* (left) and degree of linear polarization *P*_*L*_ (right) as functions of λ across the O_2_ A-band for a model planet with a surface albedo of 0.6, without a cloud (green lines) or with a cloud with optical thickness 5.0 at different altitudes. The atmosphere consists of 5 layers with gaseous scattering optical thicknesses equal to(bottom to top): 0.01, 0.003, 0.005, 0.005, 0.002. The ‘low cloud’ (orange line) is in layer 3, and the ‘high cloud’ (blue line) in layer 5. (Online version in colour.)

The presence of abundant atmospheric oxygen (O_2_) in thermodynamic disequilibrium is thought to be a robust biosignature, as on Earth, the dominant source of O_2_ is oxygenic photosynthesis [28]. Thus, an O_2_-rich atmosphere could indicate the presence of photosynthetic organisms. An exoplanet’s O_2_ mixing ratio could be derived from the depth of absorption bands in *F* and *P*_*L*_ spectra, of which the A-band, centred around 760 nm is the least contaminated by absorption lines of water [13]. This depth, however, also depends on the presence of clouds: on Earth, measurements of *F* across the A-band are routinely used to determine cloud-top altitudes [33,34], as the band depth increases with the amount of O_2_ above the clouds, and thus with a decreasing cloud-top altitude. This is evident in the *F* and *P*_*L*_ spectra across the band shown in figure 3, computed according to [26] and convolved with a Gaussian of 5 nm FWHM, informing LOUPE’s goal instrument response function (see §4.)

The lines in figure 3 were computed for a planet with a surface albedo of 0.6, completely covered by a cloud of optical thickness 5.0, and seen at *α* = 60°. It is clear that the lower the cloud, the deeper the band (with respect to the continuum) in *F*, as the more absorbing gas is above it. Because of the small atmospheric gaseous scattering optical thickness at these wavelengths, the continuum *F* is insensitive to the cloud top altitude. Note that without a cloud, the continuum *F* is higher because the cloud particles are strongly forward scattering, thus scattering light towards the surface. *P*_*L*_ is higher in the band because absorption suppresses (depolarizing) multiple scattering, and because it increases the average scattering altitude and thus the scattering by the gas, which yields a higher *P*_*L*_ at most scattering angles [26].

LOUPE’s observations will allow us to study the spectral and temporal variations of Earth’s *F* and *P*_*L*_ across the O_2_ A-band and other absorption bands, such as those of the trace gases ozone (O_3_) and water vapour (H_2_O), which are also indicative for a planet’s habitability, across a range of phase angles and with that, provide valuable insight into the diagnostic value of gaseous absorption bands in exoplanet spectra.

## 3. Scientific and technical requirements

As outlined above, the top-level science requirements for LOUPE are:

— Perform near-instantaneous (snapshot) spectropolarimetry of the entire Earth.
— Detect the presence of liquid water oceans and clouds.
— Derive and monitor atmospheric properties, e.g. via Rayleigh scattering.
— Detect the O_2_-A band in *F* and *P*_*L*_, and its variance with cloud cover and altitude, and *α*.
— Detect the Chlorophyll Green Bump and Vegetation Red Edge, the spectroscopic signature of plant life.
— Derive a map of continents from the disc-integrated signal and identify notable features, such as rain forests, deserts and ice caps.

LOUPE shall perform its science goals by recording and demodulating the disc-integrated Stokes vector of sunlight reflected from the Earth. The minimum and goal technical requirements for LOUPE’s mission are stated in table 1.

**Table 1.** Minimum and goal technical requirements for LOUPE.

requirement	minimum	goal	rationale
spectral range	500–800 nm	400–1000 nm	(Rayleigh scattering ∼400 nm,) Green bump: 500–600 nm, VRE: 700–760 nm, O_2_A band: 750–770 nm, (H_2_O bands: 800–1000 nm)
spectral resolution	20 nm	5 nm	20 nm suffices for broad spectral features, 5 nm would enable a detailed look into the O_2_A band (possible implementation as a separate channel).
polarization parameters	*F*(*λ*), *P*_*L*_(*λ*), *χ*_*L*_(*λ*)	full Stokes	Circularly polarized flux *V* could be introduced in a separate channel, using modulation optics similar to LSDpol [30].
polarimetric sensitivity	0.1%	<10^−4^	The smallest detectable change in fractional polarization. Must be able to detect the weaker features in figure 1, e.g. Green Bump, O2A band [13].
polarimetric accuracy	1%	<10^−3^	Accuracy of measurements limited by systematic errors. Planetary properties affect the polarization signal at below 1% level [25].
relative photometry	3%	1%	To track the diurnal changes in flux with a sufficient SNR [5].
radiometric accuracy	no requirement	∼1%	Retrieval accuracy for the absolute value of flux. Goal derived from NISTAR radiometric instrument aboard the DSCOVR mission for potential climate research applications [20].
field of view/pointing	20° × 20°, rough pointing	avoidance of horizon and Sun, active pointing	Diameter of Earth is 2°, Lunar libration ±8°. In the case of polar lander, ensure Earth is in FOV *a priori* in the passive pointing scenario, *a posteriori* with active pointing.
spatial sampling	unresolved	continent sized	Measurements must enable easy disc-integration of signal.
temporal sampling	hourly	multiple/hour	To capture the Earth’s diurnal rotation and enable continent mapping. Minimum derived from EPIC imager aboard the DSCOVR mission [21].
mission duration	1 month	1 + years	To get an overview of phase angles, and additionally the seasonal variation in polarized flux.
mass	<1 kg	300 g	Ensure minimal addition to payload mass. Includes electronics and protective mechanism.
volume	1 U	<1 U	Ensure versatility for potential 1U CubeSat proof of principle.
moving parts	protective lid (single use)	active pointing and protection	Active protection from the Solar glare or Lunar dust would improve data quality over data-driven masking.
data bandwidth	∼50 MB/day	>100 MB/day	One compressed observation is expected to produce 2 MB of data. Temporal sampling will determine total daily load.

In order to maximize deployment opportunities, LOUPE will be prototyped with platform versatility in mind, so that it may be suitable for multiple use cases (including geostationary, Lunar orbiting and landing scenarios). To keep a first proof-of-concept version of LOUPE as simple as possible, key trade-offs may be undertaken, such as limiting the instrument not to measure the circular polarization *V*, but only *F*, *Q* and *U*. This trade-off is justified by the fact that, although *V* has the potential to be considered a biosignature of homochiral life [29], *V* of light reflected by an (exo)planet is several orders of magnitude lower than the linearly polarized flux [25,35,36] and neglecting it introduces no significant errors in *F*, *Q* and *U* [37]. Some other capabilities of LOUPE are functionally optional as well, such as radiometry, though its addition could provide data for climate research. The additional capability to resolve the Earth at the continent scale would also introduce additional signal processing difficulties, as the ability to perform straightforward disc-integration of the signal is integral to LOUPE’s mission. Weighing the advantage of a wide field of view with passive pointing against a narrower, baffled field of view with better protection from the Solar glare, but a need for active pointing, is another topic for contemplation in the design process.

## 4. LOUPE instrument design

**Figure 4.**
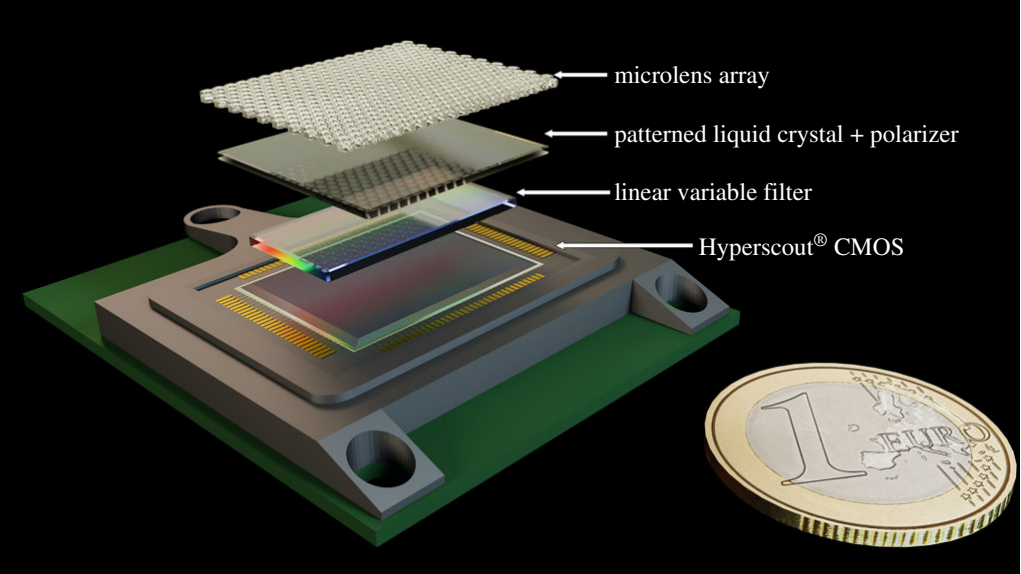
A three-dimensional render of the current LOUPE concept, with a € 1 coin for scale. (Online version in colour.)

The leading instrument design principle adopted for LOUPE is to create a compact, low-mass, low-volume, space-ready hyperspectropolarimeter with no moving parts [5]. These constraints require creative solutions from the cutting edge of hyperspectral^4^ and polarimetric instrument design, where polarimeters traditionally used active rotating optics (temporal modulation) or beam-splitting (spatial modulation) [38–40]. Since the first design iteration and proof-of-concept study of LOUPE [6], further improvements forgo the use of bulky imaging and reimaging optics, resulting in a compact, solid-state instrument with a novel approach to snapshot spectropolarimetry. Figure 4 shows a tentative 3D-render of LOUPE’s latest design.

**Figure 5.**
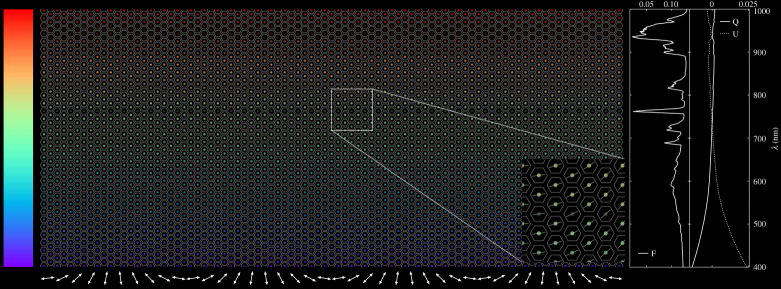
A simulated LOUPE detector snapshot. Each coloured dot is an unresolved Earth-image, filtered spectrally along the vertical (denoted by colour), with polarization modulation along the horizontal (denoted by the arrows). The wavelength dependence of *F*, *Q* and *U* is plotted on the right-hand side. The input spectrum is a simulated fully cloudy planet, with the spectral resolution set to approximately 3 nm, and the detector is rotated by 30° with respect to the planetary scattering plane. (Online version in colour.)

The challenge for LOUPE is collapsing four-dimensional data (*F*, *P*, *χ*, and λ) onto a two-dimensional detector, instantaneously for Earth’s entire disc. The instrument will be built on cosine Remote Sensing’s^5^ HyperScout^®^^6^ hyperspectral imaging platform [41,42], space qualified and operating in Earth orbit for almost two and a half years,^7^ which is based on CMOS and linear variable filter (LVF) technologies. Because apart from the Sun, the Earth is the brightest object in the sky as seen from the Moon, we can use a wide-field micro-lens array (MLA) instead of a traditional telescope objective system. Each ‘fisheye’ MLA-lenslet focuses the Earth as a dot on the detector (figure 5). We therefore forfeit the ability to resolve features on the Earth’s disc, in favour of recording an unresolved point source, similar to observations of distant exoplanets.

By overlaying a linear variable filter (LVF) atop the detector, every pixel will be filtered spectrally in the direction of the LVF gradient (figure 5). The polarization information is encoded in the perpendicular direction (figure 5) using a technique of cross-spectral modulation analogous to the rotating retarder polarimeter [30,31]. Uniquely to this design, and similarly to that of LOUPE’s ‘cousin’, LSDpol, this is achieved by placing a linear polarizer and a patterned liquid crystal (PLC)[43] on top of the spectrometer. The liquid crystal pattern is such that it behaves as an achromatic half-wave plate [44] for all wavelengths of interest. This combination of polarizer and PLC acts as a passive modulator superimposing a sinusoidal modulation on the flux spectrum in the cross-spectral direction. This modulation has the same form as the case of a rotating wave plate polarimeter described in [38], and ensuring several modulation cycles across the detector provides redundancy in case of bad pixels or local dust accumulation. The amplitude of this modulation scales with *P*_*L*_, and its phase with *χ*. By demodulating the signal in post-processing, the full polarization information can be retrieved in parallel with a spectral measurement at full spectral resolution. Additional resolution across spectral regions of interest, e.g. the O_2_ A-band, can be achieved by installing ring resonators as bandpass filters on separate ‘pixels’ next to the HyperScout^®^ focal plane.

In conclusion, a LOUPE observation will consist of an array of ‘pale (blue) dots’ in all colours of the spectrum, modulated with respect to angle and degree of polarization. We can then extract the disc-integrated Stokes vector by demodulating this two-dimensional array of dots, and proceed to compare it to our numerically simulated planet signals. The features we identify in our analysis can be verified by comparison to satellite data.

Another benefit of this compact design is that the need for instrument pointing has been effectively removed. The offset of an Earth-dot from its respective lenslet centre in figure 5 is directly related to the incidence angle of the Earth-light, enabling the retrieval of Earth’s position relative to the detector *a posteriori*. This is crucial for both the spectral and the polarization pre-flight calibration, which strongly depend on the incidence angle. In addition to the data-driven calibrations enabled by LOUPE’s elegant design, vicarious calibrations can be performed using, e.g. bright starlight of known properties. Furthermore, any persistent features caught in the instrument’s field of view—such as the Lunar surface—can be corrected for. As long as LOUPE has a direct line of sight to Earth, even accounting for Lunar libration, active mechanical pointing is not required. Yet another benefit of the MLA-design is redundancy: bad pixels or lenses covered by Lunar dust can be corrected for in post-processing.

The preliminary design fits well within the dimensions of 1 U and *ca* 300 g (table 3), and can be adapted to a variety of landing, roving or orbiting missions. For instance, for a roving mission to the Lunar south pole, where the Earth remains close to the horizon, fold mirrors can be installed to ensure an image of the Earth is reflected onto the horizontal LOUPE detector without actively pointing the instrument. Alternatively, installing multiple LOUPEs so that they face various directions and span the sky might be the solution for a lander or an orbiting platform such as the Lunar Gateway.^8^

As such, LOUPE’s lightweight and robust design is a low-cost addition to any existing Lunar landing or roving mission with minimal impact to payload mass, power consumption and down-link load, as each hourly observation is expected to produce 2 MB of data at an estimated 1 kJ per image.

## 5. Conclusion

In the quest to characterize terrestrial exoplanets, the first step is an introspective look at Earth as our benchmark. The Lunar Observatory for Unresolved Polarimetry of the Earth (LOUPE) applies pioneering hyperspectropolarimetric techniques to observe Earth as an exoplanet from the Moon. LOUPE’s science mission is to guide future exoplanet observing campaigns by offering improved models of exoplanetary flux and polarization spectra, including the ability to recognize features such as clouds, continents, oceans, vegetation and oxygen abundance on worlds we cannot resolve past a single pixel. LOUPE’s novel design is being prototyped around state of the art patterned liquid crystal optics for polarimetry, working in tandem with the cosine HyperScout^®^ hyperspectral imager for spectroscopy, which was first launched to orbit in 2018. Following design, manufacturing, testing and calibration, the first flight qualified model of LOUPE is expected to be ready in 2022, resulting in a compact, light-weight addition to any mission orbiting or landing on the near side of the Moon.

## Footnotes

1 NASA’s PACE mission, that is planned for launch at the end of 2022, will carry two polarimeters: SPEXone and HARP2.

2 Accounting for the approximate 8° apparent motion of the Earth on the celestial sphere due to the Lunar libration.

3 The range of phase angles *α* an exoplanet can attain is 90° ≤ *α* ≤ 90° + *i*, with orbital inclination angle *i* equal to 0° (90°) for a face-on (edge-on) orbit.

4 Unlike traditional digital imaging, which records each pixel value in three discrete bands of red, green and blue visible light, spectral imaging records each pixel spectrum in a larger number of wavelength bands, possibly extending beyond the visible. Accordingly, hyperspectral imaging records spectra in continuous spectral bands with a very fine wavelength resolution.

5 See https://www.cosine.nl/.

6 See https://hyperscout.nl/.

7 See http://www.esa.int/ESA_Multimedia/Images/2020/05/HyperScout_view_of_Netherlands.

8 See http://www.esa.int/Science_Exploration/Human_and_Robotic_Exploration/Exploration/Gateway.

## Data accessibility

This article has no additional data.
